# LRRC8A is dispensable for a variety of microglial functions and response to acute stroke

**DOI:** 10.1002/glia.24156

**Published:** 2022-02-12

**Authors:** James R. Cook, Anna L. Gray, Eloise Lemarchand, Ingo Schiessl, Jack P. Green, Mary C. Newland, Douglas P. Dyer, David Brough, Catherine B. Lawrence

**Affiliations:** ^1^ Geoffrey Jefferson Brain Research Centre, Manchester Academic Health Science Centre Northern Care Alliance NHS Group, University of Manchester Manchester UK; ^2^ Division of Neuroscience and Experimental Psychology, Faculty of Biology, Medicine and Health University of Manchester Manchester UK; ^3^ Wellcome Centre for Cell‐Matrix Research, Lydia Becker Institute of Immunology and Inflammation, Division of Infection, Immunity and Respiratory Medicine, Faculty of Biology Medicine and Health, University of Manchester Manchester UK

**Keywords:** cerebral ischaemia, chemotaxis, microglia, motility, phagocytosis, VRAC

## Abstract

Microglia, resident brain immune cells, are critical in orchestrating responses to central nervous system (CNS) injury. Many microglial functions, such as phagocytosis, motility and chemotaxis, are suggested to rely on chloride channels, including the volume‐regulated anion channel (VRAC), but studies to date have relied on the use of pharmacological tools with limited specificity. VRAC has also been proposed as a drug target for acute CNS injury, and its role in microglial function is of considerable interest for developing CNS therapeutics. This study aimed to definitively confirm the contribution of VRAC in microglia function by using conditional LRRC8A‐knockout mice, which lacked the essential VRAC subunit LRRC8A in microglia. We demonstrated that while VRAC contributed to cell volume regulation, it had no effect on phagocytic activity, cell migration or P2YR12‐dependent chemotaxis. Moreover, loss of microglial VRAC did not affect microglial morphology or the extent of ischemic damage following stroke. We conclude that VRAC does not critically regulate microglial responses to brain injury and could be targetable in other CNS cell types (e.g., astrocytes) without impeding microglial function. Our results also demonstrate a role for VRAC in cell volume regulation but show that VRAC is not involved in several major cellular functions that it was previously thought to regulate, and point to other, alternative mechanisms of chloride transport in innate immunity.

## Main Points

- LRRC8A KO does not affect microglial phagocytosis or migration.
- LRRC8A does not regulate microglial P2RY12‐dependent chemotaxis or morphology.
- Microglial LRRC8A does not affect ischaemic stroke damage.

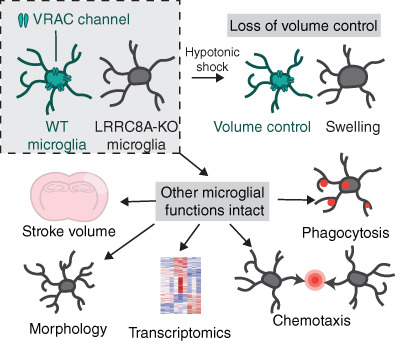

## 1 INTRODUCTION

Microglia are resident innate immune cells in the central nervous system (CNS), and are critical in orchestrating inflammatory responses to brain injury (such as stroke or trauma) and limiting the spread of damage (Hanisch & Kettenmann, 2007; Li & Barres, 2018). To this end, microglia are capable of sensing insults, migrating toward injured regions, phagocytosing dying cells, debris and pathogens, as well as initiating inflammatory responses via secretion of cytokines (Hanisch & Kettenmann, 2007; Li & Barres, 2018). Ion channels, including the volume‐regulated anion channel (VRAC), are implicated in regulating microglial function in both physiological and pathological settings (Kettenmann et al., 2011).

VRAC is activated in response to osmotic swelling, where it mediates chloride efflux from the cytoplasm, which is followed by osmotically obliged water in a process known as regulatory volume decrease (RVD; Osei‐Owusu et al., 2018). Leucine‐rich repeat containing 8A (LRRC8A/SWELL1), along with its paralogues LRRC8B‐E, are the molecular components of VRAC (Qiu et al., 2014; Voss et al., 2014). VRAC channels are heterohexameric assemblies, with at least one LRRC8A subunit required for activity (Osei‐Owusu et al., 2018). LRRC8A alone, however, cannot form functional VRACs and requires the presence of at least one of the other four paralogues (Osei‐Owusu et al., 2018). Beyond these basic constraints, subunit composition is variable, and differing stoichiometries determine the electrophysiological characteristics and solute specificities of the resulting channel (Lutter et al., 2017; Schober et al., 2017). In addition to chloride, VRAC transports a large repertoire of substrates, which are generally small organic osmolytes. Examples include amino acids such as glutamate and aspartate, as well as nucleosides such as ATP and cyclic GMP‐AMP (cGAMP; Lahey et al., 2020; Osei‐Owusu et al., 2018; Zhou et al., 2020a). As such, VRACs are capable of influencing not only cell volume, but also various intercellular signaling pathways.

VRAC inhibitors such as tamoxifen, 5‐nitro‐2‐(3‐phenylpropyl‐amino) benzoic acid (NPPB) and 4‐(2‐butyl‐6,7‐dichlor‐2‐cyclopentylindan‐1‐on‐5‐yl) oxobutyric acid (DCPIB) are known not only to inhibit RVD, but also cell migration and phagocytosis, which are critical to microglial and macrophage function (Ducharme et al., 2007; Furtner et al., 2007; Harl et al., 2013; Schwab et al., 2012; Zierler et al., 2008). Additionally, VRAC blockers inhibit ATP‐dependent chemotaxis of microglial processes following laser ablation in brain slices and prevent ramification of microglia in vitro (Eder et al., 1998; Hines et al., 2009). Thus, it has been suggested that VRAC‐dependent chloride conductance also serves to regulate cell shape changes such as those required for ramification, migration and engulfment of particles. However, interpretation of these results with respect to VRAC is complicated by the limited specificity of chloride channel blockers for VRAC. Genetic studies are therefore required to confirm whether VRAC regulates these critical activities.

Astrocytic VRACs have been shown to act as glutamate release channels, regulating both baseline neural excitability and excitotoxic injury following cerebral ischemia (Yang et al., 2019; Zhou et al., 2020b). Mice with astrocyte‐specific or whole‐brain deletion of LRRC8A develop significantly smaller infarcts in experimental stroke models (Yang et al., 2019; Zhou et al., 2020b). Thus, VRAC channels could serve as a target for reducing acute neurotoxicity following CNS injury. However, pharmacological VRAC inhibition is likely to be indiscriminate of cell type and could potentially impair protective microglial functions. As such, greater understanding of the role of VRAC in microglial physiology is required, particularly in the context of brain injury.

To determine the true contribution of VRAC to microglial function, we utilized microglia/macrophage‐specific LRRC8A knockout mice. In this model, we demonstrate that microglial development, morphology, and function remain intact despite complete loss of volume control in response to hypo‐osmotic stress. Moreover, we show that microglial VRAC did not influence infarct volume in cerebral ischemia. Thus, we conclude that VRAC channel activity is dispensable for numerous important aspects of microglial physiology.

## 2 MATERIALS AND METHODS

### 2.1 Chemicals

DCPIB (Tocris), NPPB (Tocris), FFA (Sigma), Cytochalasin D (Sigma) and Calcein‐AM (Biolegend) were obtained as powders dissolved in DMSO to ×200 concentrated stocks and stored at −20°C. pHrodo‐SE (ThermoFisher) was dissolved in DMSO to 10 mg/ml and stored in aliquots at −80°C. Human Aβ_1‐42_ (Eurogentec) was dissolved in hexafluoro‐2‐propanol, dried into films under a rotary evaporator (Eppendorf) and stored at −80°C. Tomato lectin conjugated to DyLight 594 (Vector Biolabs) was washed twice through a centrifugal filter (10 kDa MWCO) to remove sodium azide, sterile filtered and stored as a 1 mg/ml stock at 4°C.

### 2.2 Animals

The *Lrrc8a* ^*fl/fl*^ line was generated in‐house using CRISPR‐Cas9 as described previously (Green et al., 2020) and crossed with a *Cx3cr1* ^*Cre*^‐expressing line (Yona et al., 2013) to generate littermates homozygous for the floxed LRRC8A allele and either null (*Lrrc8a* ^*fl/fl*^ *:Cx3cr1* ^*+/+*^ *)* or heterozygous (*Lrrc8a* ^*fl/fl*^ *:Cx3cr1* ^*+/Cre*^ *)* for *Cx3cr1* ^*Cre*^, corresponding to WT and KO groups, respectively. The Cre‐expressing line (*Lrrc8a* ^*wt/wt*^ *:Cx3cr1* ^*+/Cre*^) were also used in some studies as controls for Cre. All animals were housed under standard conditions (21 ± 2°C; 55% ± 5% humidity) in individually ventilated cages, under 12‐h light/dark cycles with ad libitum access to food and water. Unless otherwise specified, all animals were at least 8 weeks old and of mixed sex. All in vivo work was performed in accordance with the Animals (Scientific Procedures) Act 1986 under relevant UK Home Office licenses and approved by the local Animals Welfare and Ethical Review Board (University of Manchester, UK).

### 2.3 Cell culture

For microglia cultures, adult mice (8–44 weeks old) were perfused with ice‐cold saline and the brains collected, the cerebellum and olfactory bulb were discarded, and the remaining tissue diced into small (1–2 mm) chunks using a scalpel. Papain and DNAse digestions were performed using a neural tissue dissociation kit (Miltenyi) according to the manufacturer's instructions. Following digestion, a dounce homogenizer with a loose pestle was used to dissociate the brain into a single‐cell suspension, which was then pelleted at 300*g* (5 min) and myelin removed by centrifugation through 30% Percoll Plus (GE Healthcare). Cells were then resuspended in MACS buffer (PBS, 0.5% BSA, 2 mM EDTA) with anti‐CD11b magnetic beads (Miltenyi) and incubated on a roller for 15 min. Microglia were enriched using a quadroMACS separator with LS columns (Miltenyi) according to the manufacturer's instructions. After counting, 1–2 x 10^4^ cells were spot‐plated onto 96‐well Cell+ anionic/cationic plates (Sarstedt) and allowed to adhere for 10 min before flooding with media. Cells were cultured for at least 7 days in DMEM/F12 (Gibco) supplemented with 1% penicillin/streptomycin (Sigma), 2 mM glutamine, 10% FBS (Gibco), 50 ng/ml TGF‐β2 (Peprotech) and 20 ng/ml IL‐34 (R&D Systems).

Bone marrow‐derived macrophages (BMDM) were cultured by harvesting bone marrow from both femurs of each mouse, lysing red blood cells in ACK buffer, and culturing for 7 days in DMEM (10% FBS, 100 U/ml penicillin, 100 μg/ml streptomycin) mixed 70:30 with DMEM conditioned by L929 fibroblasts.

### 2.4 Western blotting

For western blots, cells were lysed directly in laemmli buffer (62.5 mM Tris–HCl, 2% SDS, 10% glycerol, 5% β‐mercaptoethanol) and heated to 95°C for 10 min. Lysates were then separated by tris‐glycine SDS‐PAGE before transfer to PVDF membranes. Membranes were blocked in 5% nonfat milk in phosphate‐buffered saline (PBS) with 0.05% Tween‐20 (PBS‐T) for 1 h at room temperature before incubating overnight with mouse anti‐LRRC8A (8H9, Santa Cruz, 1:100) in block buffer. Membranes were washed three times in PBS‐T before incubating with HRP‐conjugated rabbit anti‐mouse (Dako) in block buffer for 1 h at room temperature. After washing three further times, membranes were imaged on a G:Box Chemi XX6 (Syngene) using Amersham ECL Prime chemiluminescence reagent (GE Healthcare).

For loading controls, membranes were washed and incubated with HRP‐conjugated mouse anti‐β‐actin (Sigma) in block buffer for 1 h at room temperature, followed by washing and imaging as above.

### 2.5 Regulatory volume decrease assay

5 x 10^4^ BMDM were seeded onto black‐walled 96‐well plates (Corning), whereas microglia were spot‐plated onto Cell+ plates. Cells were loaded with 10 μM calcein‐AM (BioLegend) for 1 h, washed three times with media and then rested for 30 min to allow calcein to equilibrate. After three further washes with isotonic buffer (132 mM NaCl, 2.5 mM KCl, 2 mM CaCl_2_, 2 mM MgCl_2_, 10 mM Glucose, 20 mM HEPES, pH 7.4, 312 mOsm/L) cells were imaged on an Eclipse Ti microscope (Nikon) equipped with a stage incubator maintaining 37°C and 5% CO_2_. Calcein fluorescence was captured in the GFP channel using low laser power to minimize phototoxicity and bleaching. Live imaging was conducted using point‐visiting to image all conditions simultaneously. One image was captured every 2 min, with hypotonic shock induced at 5 min by adding distilled water. For quantification, fluorescent images were processed in Fiji, using a rolling‐ball background subtraction (50 pixels ball size) to remove non‐cell associated fluorescence. The average pixel intensity of each frame was then measured and normalized to the value of the first frame, yielding *F*/*F*_0_ curves. For statistical testing, AUC values of the curves were calculated in Prism.

### 2.6 Phagocytosis assays

Phagocytosis assays were performed using an IncuCyte ZOOM (Essenbio) time‐lapse microscope housed in a humidified incubator maintaining 37°C and 5% CO_2_. Prior to imaging, cells were incubated in Opti‐MEM (containing drugs or vehicle as specified) for 15 min. pHrodo‐*Escherichia coli* bioparticles (ThermoFisher) and pHrodo‐Zymosan (ThermoFisher) were then added at a 1:10 dilution. Other prey particles were prepared and added as described below.

Human Aβ_1‐42_ (Eurogentec) was dissolved to 10 mM in DMSO, then further diluted to 1 mM using sterile water and incubated at 37°C for 7 days. Fibrils were pelleted by centrifugation at 18,000*g* for 15 min, labeled with 25 μM pHrodo red‐SE (ThermoFisher) in PBS at room temperature for 1 h, washed twice with PBS and added to cells at a final concentration of 10 μM.

For RBC assays, whole mouse blood was collected by cardiac puncture and centrifuged briefly at 500*g* to pellet cells. The pellet was then resuspended in PBS, layered onto a discontinuous percoll gradient containing 65% and 35% layers and centrifuged at 1000*g* for 15 min. RBCs were recovered from the pellet, washed once with PBS, and 10^8^ cells were labeled with 50 μM pHrodo‐SE for 1 h, followed by two washes with PBS. Labeled RBCs were then opsonized with 1 μg/ml rabbit anti‐mouse RBC (34‐3C, Hycult) for 30 min, washed two further times with PBS, and added to cells at 10^6^ RBC per well. Un‐opsonised controls were included to verify that uptake was IgG‐dependent.

Following addition of particles, three images per well were captured every 15 min for a total of 3 h, and automatically analyzed using the IncuCyte software (Essenbio). Custom scripts were created which gave optimal detection for each phagocytic substrate and were run identically on all conditions. For each well, the pHrodo‐positive area was normalized to the area covered by cells (determined from the baseline image using phase confluence function) to give the phagocytic index.

### 2.7 In vitro motility assay

Microglia cultured in 96‐well plates were labeled with Hoechst (1 μg/ml) in growth media for 45 min, washed with serum‐free media and incubated in Opti‐MEM (containing vehicle or drugs as stated) for 15 min prior to imaging. Cells were imaged on a Nikon Eclipse Ti widefield microscope with a stage incubator maintaining 37°C/5% CO_2_ using both phase contrast and DAPI channels with low UV laser power to prevent toxicity. Images were obtained every 5 min for 3 h.

The fluorescent (nuclear) channel was then analyzed for cell movement in ImageJ by first performing a rolling‐ball background subtraction (ball radius 20) and loading the resulting images into the TrackMate plugin (Tinevez et al., 2017). To identify nuclei, LoG detection was used with a spot size of 8 μm, thresholds were empirically determined for each experiment and applied equally across all conditions. No spot filtering was necessary. Tracking was performed using the simple LAP tracker with max linking distance of 40 μm, and max gap closing distance of 70 μm over two frames.

### 2.8 RNA sequencing

Microglia were purified from 10–12 week‐old female WT, KO and Cre mice via MACS and RNA isolated immediately using Purelink RNA miniprep kits (Invitrogen). Total RNA was submitted to the genomic technologies core facility (GTCF) at the University of Manchester. Quality and integrity of the RNA samples were assessed using a 2200 TapeStation (Agilent Technologies) and then libraries generated using the TruSeq® Stranded mRNA assay (Illumina, Inc.) according to the manufacturer's protocol. Briefly, total RNA (0.1–4 ug) was used as input material from which polyadenylated mRNA was purified using poly‐T, oligo‐attached, magnetic beads. The mRNA was then fragmented using divalent cations under elevated temperature and then reverse transcribed into first strand cDNA using random primers. Second strand cDNA was then synthesized using DNA Polymerase I and Rnase H. Following a single “A” base addition, adapters were ligated to the cDNA fragments, and the products then purified and enriched by PCR to create the final cDNA library. Adapter indices were used to multiplex libraries, which were pooled prior to cluster generation using a cBot instrument. The loaded flow‐cell was then paired‐end sequenced (76 + 76 cycles, plus indices) on an Illumina HiSeq4000 instrument. Finally, the output data was demultiplexed (allowing one mismatch) and BCL‐to‐Fastq conversion performed using Illumina's bcl2fastq software, version 2.20.0.422.

For analysis, unmapped paired‐end sequences were tested by FastQC (https://www.bioinformatics.babraham.ac.uk/projects/fastqc/). Sequence adapters were removed and reads were quality trimmed using Trimmomatic_0.36 (Bolger et al., 2014). The reads were mapped against the reference mouse genome (mm10/GRCm38) and counts per gene were calculated using annotation from GENCODE M21 (https://www.gencodegenes.org/) using STAR_2.5.3a (Dobin et al., 2013). Normalization, Principal Components Analysis, and differential expression was calculated with DESeq2_1.28.1 (Love et al., 2014). Differentially expressed genes between conditions (Log2 fold‐change >0.5, adjusted *p*‐value <.01) were tested for pathway enrichment using the enrichR platform (Chen et al., 2013) queried via the enrichR package (https://CRAN.R-project.org/package=enrichR) in Rstudio.

### 2.9 RT‐qPCR

RNA was isolated using PureLink kits (Invitrogen) and cDNA transcribed using SuperScript III first‐strand synthesis kits (Invitrogen). RT‐qPCR was performed using 5 ng cDNA along with 200 nM forward and reverse primers (Table 1) mixed with SYBR Green mastermix according to the manufacturer's protocol. All samples were loaded in triplicate wells of a MicroAmp 384‐well optical PCR plate (Applied Biosystems) and immediately assayed on a 7900HT Fast Real‐Time PCR machine (Applied Biosystems) for 40 cycles with standard settings. For each primer pair, a standard curve consisting of four 10‐fold dilutions of neat cDNA was run in parallel to determine amplification efficiency. Expression values of Lrrc8a exons were first normalized to Gapdh values as a loading controls, and Gapdh‐normalized expression of exon 3 was further normalized to expression of exons 1–2 in the same sample to account for varying baseline *Lrrc8a* expression levels.

**TABLE 1:** Primers used for RT‐qPCR analysis

*Lrrc8a* (exon 1–2)	Forward	GAGCAAAAGGAATGTCAGGGC
	Reverse	TCTTTGTGTTGGCTGTTGGTG
*Lrrc8a* (exon 1–2)	Forward	ACATCCCCGACGTCAAGAAC
	Reverse	GCGCAGCTTGTTTTCACTCA
*Gapdh*	Forward	CAGTGCCAGCCTCGTCC
	Reverse	CAATCTCCACTTTGCCACTGC

*Note*: All sequences are given in the 5′‐3′ direction.

### 2.10 Acute brain slices

Acute brain slices were prepared according to published methods (Etienne et al., 2019). Briefly, adult (8–24 week‐old) Cx3cr1‐eGFP mice were perfused with ice‐cold oxygenated choline‐artificial cerebrospinal fluid (aCSF; 110 mM choline chloride, 25 mM glucose, 25 mM NaHCO_3_, 20 mM HEPES, 0.5 mM CaCl_2_, 7 mM MgCl_2_, 11.6 mM ascorbic acid, 3.1 mM sodium pyruvate, 2.5 mM KCl, 1.25 mM NaH_2_PO_4_, pH 7.4), the brain was quickly dissected, and 300 μm‐thick slices cut on a VT1000S vibratome (Leica). Slices were allowed to recover in choline‐aCSF at 34°C for 15 min under constant oxygenation before transfer to experimental aCSF (124 mM NaCl, 25 mM glucose, 25 mM NaHCO_3_, 2 mM CaCl_2_, 1 mM MgCl_2_, 2.5 mM KCl, 1.25 mM NaH_2_PO_4_) at room temperature bubbled constantly with carbogen. Slices were used between 40 and 180 min after first being cut. For imaging, slices were mounted in a heated tissue chamber (Scientifica) maintained at 37°C and constantly perfused with carbogenated aCSF via a peristaltic pump with inline heater. Imaging was performed at least 50 μm below the surface of the slice to avoid reactive microglia.

### 2.11 Lectin injection and cranial window implantation

Cranial windows were implanted as previously described (Goldey et al., 2014). Animals were anesthetized with 2.5% isoflurane in 100% O_2_ and the scalp was removed over the animals left and right hemisphere. A metal head plate was mounted (Narishige CP‐2, Japan) using dental cement (Sun Dental, Japan) to allow stereotaxic fixation under the 2‐photon microscope. A circular piece of bone with a diameter of 3 mm was then removed using a dental drill. The dura was left intact for the whole experiment. For this experiment, animals with body weights between 20 and 25 g were used—generally males were 8–12 weeks old and females were 8–20 weeks old.

Prior to window implantation, tomato lectin conjugated to DyLight‐594 was diluted to 50 μg/ml in sterile PBS, and 200 nl was injected through the dura into the cortex using a borosilicate microcapillary tube pulled to an outer tip diameter of 5 μm. Injections were performed over 10 min (UltraMicroPump III, World Precision Instruments, USA), followed by a 5 min rest before the needle was withdrawn. Injection depths were between 150 and 250 μm below the dura. Once the injection was finished, a circular coverslip (Warner Instruments, USA) was glued in place of the removed bone using dental cement (Sun Dental, Japan).

Mice were imaged immediately after cranial window implantation over a period of 4 h. During this time, they were maintained under 2% isoflurane in 100% O_2_ and the body temperature was maintained at 37.5°C via a heating blanket controlled with a temperature probe (Harvard Apparatus, Kent, UK).

### 2.12 Multiphoton imaging

Imaging was conducted using a Leica SP8 multiphoton microscope equipped with a MaiTai Ti:Sapphire MP laser (Spectra‐Physics) and an HC Fluotar L 40× water dipping lens. 1024 x 1024 pixel z‐stacks were captured at ×1.8 confocal zoom, corresponding to approximate dimensions of 180 x 180 μm, with *Z*‐planes spaced 2 μm apart. For each series, 15–20 *z*‐planes were captured, corresponding to 15–20 μm above and below the lesion. For imaging GFP, the 2‐photon laser was tuned to 880 nm, and to 800 nm for imaging tomato lectin. In each case, imaging laser power was kept below 5% and ×3 line averaging was used. Lesions were induced using the point bleach function in the LAS X software to illuminate a single pixel for 500 ms with laser power set to 20–30x that used for imaging. Image acquisition was then automatically started, and z‐stacks acquired 1 min apart for 10 (for Cx3cr1‐eGFP brain slices) or 15 min (in vivo imaging).

For analysis, xyzt hyperstacks were first registered in Fiji using the turboreg plugin, and then cropped to a 120 μm x 120 μm square centered around the lesion. A median filter with a radius of 2 pixels was then applied, and background subtraction with a rolling ball radius of 50 pixels performed. Maximum‐intensity z‐projections were then created and a further median filter applied to smooth noise. eGFP images were then thresholded using the Huang method. For tomato lectin images, thresholds were empirically determined due to variability in the staining intensity and presence of other bright objects (e.g., blood vessels), which complicated automatic thresholding. Thresholded time‐series were then analyzed in MATLAB using the MGPtracker script (Gyoneva et al., 2014; available from the original authors upon request), which quantifies the area of a polygon in each image surrounding the lesion which is free of microglial processes. The resulting area‐over‐time data were then normalized to the value of the first frame for each video.

### 2.13 Middle cerebral artery occlusion

Thrombi formation and cerebral ischemia were performed using the FeCl_3_ method as described previously (Le Behot et al., 2014). 12–14 week‐old male *Lrrc8a* ^*fl/fl*^ *:Cx3cr1* ^*+/+*^ (WT), *Lrrc8a* ^*fl/fl*^ *:Cx3cr1* ^*+/Cre*^ (KO) for *Cx3cr1* ^*Cre*^, and *Lrrc8a* ^*wt/wt*^ *:Cx3cr1* ^*+/Cre*^ (Cre; *n* = 5–8/group) mice were anesthetized with 5% isoflurane, placed in a stereotaxic device, and maintained under anesthesia with 2.5% isoflurane in a 70%:30% mixture of O_2_ /N_2_O. A small craniotomy (1 mm diameter) was performed on the parietal bone to expose the right middle cerebral artery (MCA). A Whatman filter paper strip soaked in FeCl_3_ (30%, Sigma) was placed on the dura mater on top of the MCA for 10 min. Cerebral blood flow in the MCA territory was measured continuously by laser Doppler flowmetry (Oxford Optronix). Twenty‐four hours after occlusion, mice were imaged again using laser Doppler flowmetry for 5 min to confirm sustained occlusion, then culled via transcardial perfusion and brains processed for histology (infarct volume) and immunohistochemistry (for microglia on *n* = 5/group randomly selected brains). For determining infarct volumes, cryostat‐cut sections (10 μm) were stained with Cresyl violet. Infarct volume was analyzed using ImageJ and calculated as the sum of every lesion area multiplied by the distance between each section (0.5 mm). N.B one Cre mouse had partial reperfusion so was excluded from infarct volume analysis.

### 2.14 Immunohistochemistry

For microglia immunohistochemistry, animals were anesthetized with 2.5% isoflurane in 30:70 O_2_/N_2_O and transcardially perfused with cold saline solution followed by 4% PFA in 0.1M phosphate buffer. Brains were post‐fixed in 4% PFA for 24 h at 4°C, cryoprotected in 30% sucrose for 48 h and stored at −80°C until sectioning.

For density analysis, brain sections (30 μm) cut on a freezing sledge microtome were subjected to antigen retrieval in citrate buffer for 30 min, blocked in PBS containing 10% normal goat serum (Vector Biolabs) for 1 h, and incubated with rabbit anti‐Iba1 (Abcam EPR16588, 1:400) overnight at 4°C. Sections were washed three times with PBS + 0.05% Tween‐20 (PBS‐T) and incubated with Alexa‐594 goat anti‐rabbit IgG (Invitrogen, 1:500) for 1 h at room temperature, then counterstained with 4 μg/ml DAPI, washed ×3 and mounted in ProLong (Invitrogen). Whole slides were scanned on a 3D‐Histech Pannoramic‐250 microscope slide‐scanner using a ×20/0.80 Plan Apochromat objective (Zeiss) and the DAPI and TRITC filter sets. Representative regions of tissue (3 x 1.6 mm^2^ areas per animal) were exported as tiled tiffs using CaseViewer (3Dhistech) and analyzed in QuPath using a custom script to identify Iba1‐positive cells, which was run identically across all conditions. All analysis was conducted blind to animal genotype.

For morphological analyses, PFA‐perfused, post‐fixed brains from 12 to 14 week‐old male animals 24 h after MCAO were sectioned on a cryostat to 150 μm thickness. Iba1 staining was performed following published protocols (Heindl et al., 2018) with minor modifications. Briefly, sections were blocked and permeabilized overnight at room temperature in PBS with 20% DMSO, 2% normal goat serum, 1% Triton‐X100 and 0.5% gelatin from cold‐water fish skin, then stained with rabbit anti‐Iba1 (abcam EPR16588, 1:200) in the same buffer for 72 h at 4°C. Sections were washed three times for 1 h each in PBS, then incubated with Alexa‐594 goat anti‐rabbit IgG (Invitrogen, 1:500) and Hoechst (2.5 μg/ml) for 48 h in block buffer. After three further washes, sections were cleared by incubating with a sequence of increasingly concentrated fructose solutions to a final concentration of 80.2% wt/wt (SeeDB method Ke et al., 2013) for optimal confocal imaging.

### 2.15 Microglial morphology analysis

Cleared sections were imaged using a Leica TCS SP8 AOBS upright confocal with a ×63 HCX PL Apo lens at ×0.75 confocal zoom (effective magnification ×47.25). Z‐stacks were acquired at 1024 x 1024 resolution with 0.4 μm intervals, with between 70 and 90 μm total depth. Stacks were positioned immediately adjacent to the infarct border in the ipsilateral cortex and at the equivalent position in the contralateral cortex for each section. Image acquisition was performed blind to experimental group.

Z‐stacks were analyzed in MATLAB (R2018, The MathWorks, Natick, Massachusetts, USA) using the publicly available mmqt script (Heindl et al., 2018) (https://github.com/isdneuroimaging/mmqt) with the default parameters on all images. Briefly, this script first segments microglia from the 3D Z‐stacks, identifying the soma and branches. Skeleton analysis is then performed to reconstruct the skeleton of each microglia, and subsequently morphological measures are extracted using both the 3D volume and skeleton. A table of these measures is given in Table 2, where any measures referencing nodes (end‐nodes, branching nodes, etc.) refer to nodes comprising the microglial skeleton. The MATLAB outputs were analyzed in Rstudio (Rstudio Team, 2020) using R version 4.0.3 (R Core Team, 2020), whereby all parameters extracted were used to generate PCA plots, and several key parameters with high PCA loading values (sphericity, total nodes per cell and node volume) were extracted for graphing individually.

**TABLE 2:** Descriptions of features used for microglial morphology analysis

Parameter	Type	Description
Sphericity	Volumetric	3D sphericity
Circularity	Volumetric	2D circularity
Node volume	Volumetric	Node volume (75th percentile of each cell)
Total nodes	Skeleton	Total nodes per cell
Branching nodes	Skeleton	Total branching nodes (connecting to >2 other nodes) per cell
End nodes	Skeleton	Total end‐nodes (connecting to only 1 other node) per cell
Branch nodes	Skeleton	Total nodes within branches
Nodes per branch	Skeleton	Total nodes per major branch
End nodes per branch	Skeleton	Total end nodes per major branch
Branch segments	Skeleton	Total branch segments per cell
Segments per branch	Skeleton	Total branch segments per branch
Branch length skeleton	Skeleton	Total branch length (following skeleton)
Branch sinuosity	Skeleton	Branch skeleton length / air‐line length
Closeness	Centrality	Median node closeness (Graph theory concept)
Betweenness	Centrality	Median node betweenness (Graph theory concept)

### 2.16 Statistics

For all experiments, data were analyzed blinded to genotype and/or treatment and for in vivo experiments treatment groups were randomly allocated. Statistical testing was performed with individual mice as the statistical unit (biological replicates). Where measurements are taken from multiple cells per mouse, the average of these measurements was considered as one biological replicate.

Unless otherwise specified, data were plotted and analyzed in Graphpad Prism (Version 9.0). Normally distributed data (assessed via Shapiro–wilk test) are presented as mean ± standard error of the mean (SEM), and non‐normally distributed data are presented as median with interquartile range. Experiments with two groups were compared with a two‐tailed Student's *t*‐test. Comparisons between >2 experimental groups were conducted via one‐way ANOVA with Dunnet's post hoc in the case of multiple comparisons to a single control condition, or else by Tukey's post hoc. Appropriate transformations were applied in case of unequal variance between groups. Experiments with two independent variables were analyzed via two‐way ANOVA.

## 3 RESULTS

### 3.1 LRRC8A is required for regulatory volume decrease in microglia

**FIGURE 1:**
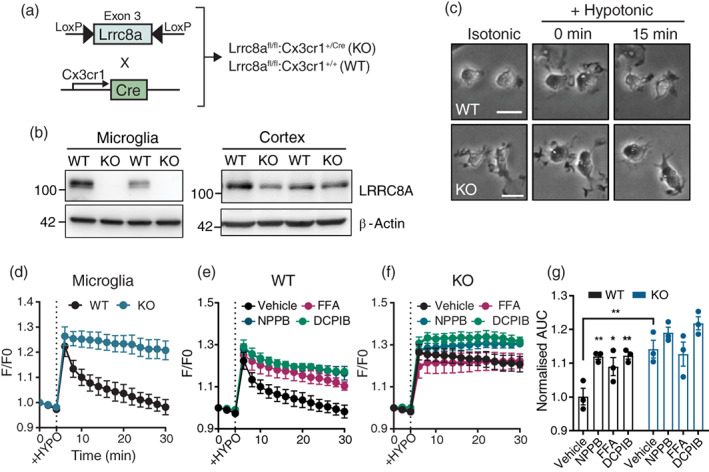
LRRC8A is required for regulatory volume decrease in microglia. (a) Schematic showing breeding strategy and genotypes of LRRC8A knock out (KO) and wild‐type (WT) mice. (b) Western blots for LRRC8A expression in isolated microglial lysates and whole‐cortex and from WT and KO mice. (c) Phase‐contrast images of WT and KO microglia under isotonic (baseline) conditions (312 mOsm/kg), followed by treatment with hypotonic (218 mOsm/kg) solution for 0 and 15 min. (d) Calcein fluorescence traces of WT and KO microglia exposed to hypotonic conditions at 5 min. (e,f) Calcein fluorescence of WT (e) and KO (f) microglia exposed to hypotonic conditions in the presence of DCPIB (10 μM), FFA (100 μM), NPPB (100 μM) or vehicle. (g) Quantification of d–f as area under curve. Data presented as mean ± SEM, from *n* = 3–4 biological replicates. Statistics in (g) are from two‐way ANOVA, *p < .05, **p < .01

In order to interrogate the role of LRRC8A in microglia/macrophage function, we generated a conditional *Lrrc8a* knockout (KO) mouse whereby transgenic mice harboring flox sequences flanking exon 3 of the *Lrrc8a* gene were crossed with the *Cx3cr1* ^*Cre*^ recombinase line (Figure 1a; Green et al., 2020). The resulting *Lrrc8a* ^*fl/fl*^ *:Cx3cr1* ^*+/Cre*^ (KO) progeny lack the major coding exon of *Lrrc8a*, resulting in complete absence of LRRC8A protein in isolated microglia, while levels in whole cortical lysate were comparable with *Lrrc8a* ^*fl/fl*^ *:Cx3cr1* ^*+/+*^ (WT) littermates (Figure 1b). To confirm that LRRC8A was functionally absent in KO cells, we performed a calcein‐based RVD assay in cultured microglia whereby changes in calcein fluorescence reflect cellular volume fluctuations. Upon exposure to a 30% hypotonic solution, WT cells underwent rapid RVD, returning to normal volume within 15 min (Figure 1c,d). By contrast, KO cells exhibited impaired RVD responses (Figure 1c,d), which were comparable to WT cells treated with the VRAC blockers NPPB, DCPIB or flufenamic acid (FFA; Figure 1e,g). These same blockers produced little additional inhibition in KO cells (Figure 1f,g), indicating that volume regulatory responses are largely dependent on LRRC8A‐containing VRACs in mouse microglia. We also confirmed that microglia and BMDM from WT (*Lrrc8a* ^*fl/fl*^ *:Cx3cr1* ^*+/+*^ *)* and Cre (*Lrrc8a* ^*wt/wt*^ *:Cx3cr1* ^*+/Cre*^) animals expressed similar levels of LRRC8A, and that WT and Cre microglia underwent RVD at a comparable rate (Figure S1).

### 3.2 LRRC8A does not affect microglial density and minimally affects gene expression

**FIGURE 2:**
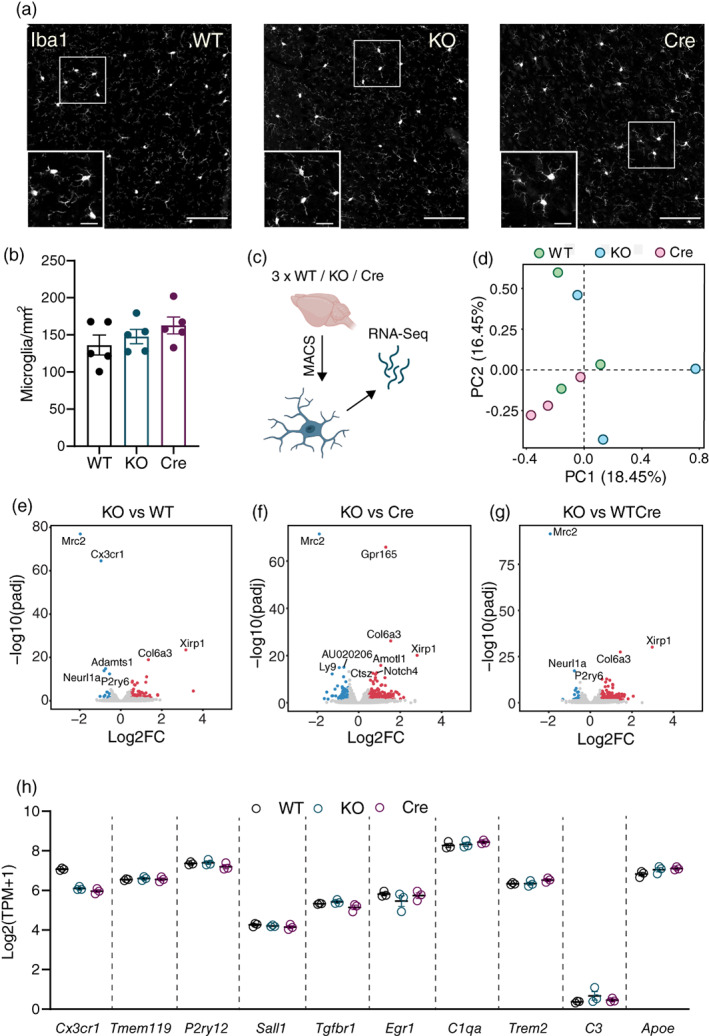
Loss of LRRC8A does not affect microglial density and minimally affects gene expression. (a) Representative photomicrographs of Iba1‐stained microglia in the cortex of wild‐type (WT), LRRC8A knockout (KO) and Cre mice, scale bars = 100 μm, inset scale bars = 25 μm. (b) Quantification of microglial density in the cortex of WT, KO and Cre mice (*n* = 5, mean ± SEM). (c) Schematic of MACS isolation and bulk RNA‐sequencing of primary acute microglia. (d) Principal component analysis of RNA‐Seq data obtained from WT, KO and Cre microglia (*n* = 3). (e–g) Volcano plots displaying upregulated (red) and downregulated (blue) genes identified between primary microglia obtained from KO and WT (e), KO and Cre (f) or KO and WT/Cre (g) animals. WT/Cre is a composite group containing both WT and Cre samples. (h) Expression of homeostatic microglial transcripts in WT, KO and Cre microglia (*n* = 3, mean ± SEM). Groups in (b) were compared via one‐way ANOVA with Tukey's post hoc

It has been reported that loss of LRRC8A can affect development and severely restrict the survival of certain cell types, including T and B lymphocytes (Kumar et al., 2014; Platt et al., 2017). To assess whether lack of LRRC8A affects microglial development or homeostasis, we first quantified microglial density in the hippocampus and cortex of WT and KO mice, as well as Cre mice to control for *Cx3cr1* hemizygosity in KO mice. All three genotypes displayed comparable numbers of microglia, indicating that LRRC8A is dispensable for microglial number and survival (Figure 2a,b). To check for differences in baseline microglial phenotype, we performed bulk RNA‐sequencing on acutely isolated microglia from adult WT, KO and Cre mice (Figure 2c). Principle component analysis revealed little consistent genotype‐dependent variability in gene expression (Figure 2d), and relatively few differentially expressed genes (DEGs) were detected when comparing KO cells to WT cells, or to a composite group combining WT and Cre cells (93 upregulated, 35 downregulated; Figure 2e–g). Upon pathway analysis, these DEGs did not appear to be enriched for any particular pathway or biological process (data not shown). Moreover, expression of the microglial homeostatic signature genes *Tmem119*, *P2ry12*, *Sall1*, *Tgfbr1* and *Egr1*, as well as Trem2 and the inflammatory markers *C3* and *ApoE* was consistent between the three genotypes (Figure 2h). *Cx3cr1* expression displayed the expected halving of expression intensity in KO and Cre microglia, but did not appear to be affected by loss of LRRC8A. Thus, lack of LRRC8A does not appear to have any profound influence on microglial survival or homeostasis at baseline.

We also considered the possibility that loss of LRRC8A might induce compensatory upregulation of other chloride channels. Analysis of the major chloride channel families expressed in microglia (Anoctamins/TMEM16, chloride intracellular channels (CLICs), voltage‐gated chloride channels (CLCNs) and Tweety channels (TTYHs)) revealed no significant alterations in any chloride channel genes between WT, KO and Cre microglia (Figure S2A). Thus, compensatory increases in other chloride channels resulting from LRRC8A disruption appear to be negligible. It is important to note that, in the RNA‐Seq dataset, *Lrrc8a* RNA was still detected in KO microglia, likely due to the fact that only exon 3 of the *Lrrc8a* gene was deleted. Indeed, qPCR using exon‐specific primers revealed intact expression of exons 1–2 in KO cells, along with almost total absence of exon 3, thus resulting in functional loss of LRRC8A protein (Figure S2B,C). The entire dataset can searched using the web‐based shinyapp (https://jrcook.shinyapps.io/MG_VRAC_RNASeq_Shiny/).

### 3.3 LRRC8A does not contribute to microglial phagocytosis

**FIGURE 3:**
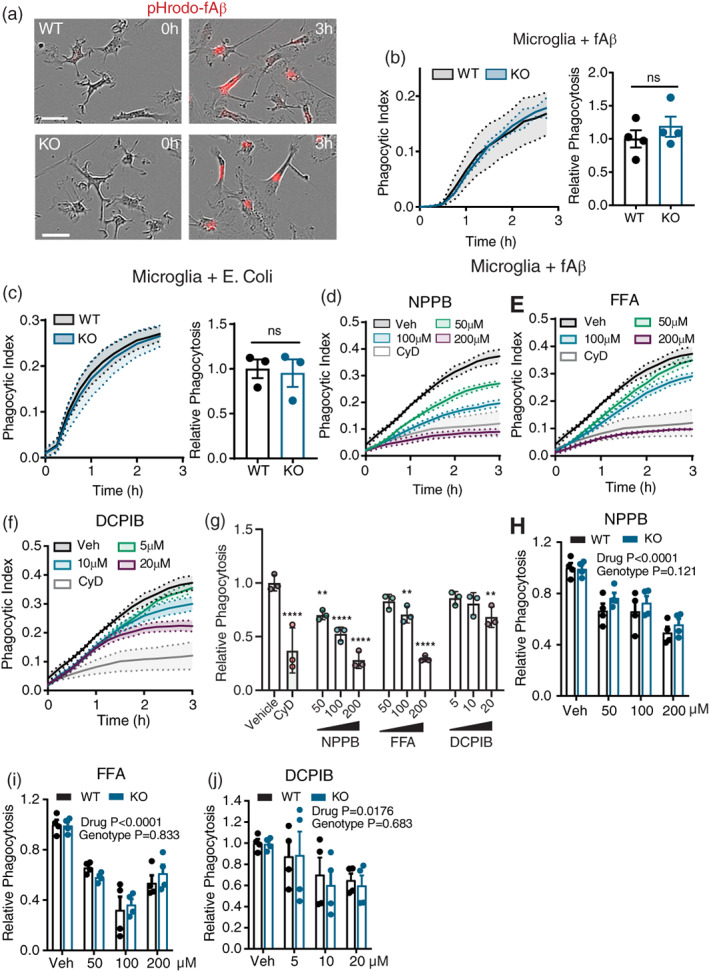
LRRC8A does not contribute to microglial phagocytosis (a) phase‐contrast and fluorescent merged images displaying uptake of pHrodo‐tagged fAβ_1‐42_ in wild‐type (WT), LRRC8A knockout (KO) microglia at 0 and 3 h post‐incubation. Scale bar = 30 μm. (b,c) Phagocytic index curves and area‐under‐curve analysis from WT and KO microglia ingesting pHrodo‐fAβ_1‐42_ (b), or pHrodo‐tagged *E. coli*‐derived bioparticles (c). (d–f) Phagocytic index curves of primary microglia ingesting pHrodo‐tagged fAβ in the presence of vehicle (Veh; DMSO), Cytochalasin D (CyD), or NPPB (d), FFA (e) and DCPIB (f). (g) AUC quantification of (d–f). (h–j) AUC‐quantified relative phagocytosis of WT and KO macrophages (BMDM) ingesting pHrodo‐*E. coli* bioparticles in the presence of varying concentrations of NPPB (h), FFA (i) and DCPIB (j). All data are presented as mean ± SEM. Phagocytic index traces are means of two biological replicates from a single experiment. AUC data points represent biological replicates and are normalized to the mean of the WT control. All experiments contain data from 3 to 6 mice. Statistics in B‐C are from two‐tailed Student's *t*‐tests, g is from one‐way ANOVA with Dunnett's post hoc and h–j are from two‐way ANOVA. **p < .01, ****p < .0001

Previous studies have indicated that VRAC blockers applied to macrophages suppress phagocytosis of latex beads and *E. coli*, implying a role for LRRC8A‐dependent volume regulation in phagocytosis (Ducharme et al., 2007; Furtner et al., 2007; Harl et al., 2013). To investigate the contribution of LRRC8A to phagocytosis in microglia we incubated primary adult microglia from WT and KO mice with phagocytic substrates tagged with pH‐sensitive pHrodo dyes and quantified phagocytic capacity via time‐lapse fluorescence microscopy (Figure 3). Microglia incubated with fibrillar amyloid‐beta_1‐42_ (fAβ_1‐42_) (Figure 3b) or *E. coli*‐derived bioparticles (Figure 3c) exhibited rapid uptake over 3 hours, and area‐under‐curve (AUC) analysis indicated comparable uptake between WT and KO cells, indicating that LRRC8A expression and volume regulatory processes are entirely dispensable for phagocytosis of a variety of substrates. We also observed that phagocytosis of fAβ_1‐42_ was dose‐dependently suppressed by NPPB, FFA and DCPIB in WT microglia (Figure 3d–g). To rule out cell‐type or substrate‐specific effects, we also confirmed these results in bone‐marrow derived macrophages (BMDM) from WT and KO animals incubated with IgG‐opsonised red blood cells, Zymosan, and *E. coli*‐derived bioparticles (Figure S3A–D). No differences in phagocytic uptake were detected for any substrate in BMDM cells, indicating that VRAC is generally dispensable for various phagocytic pathways in macrophages. While VRAC inhibitors were also effective in reducing phagocytosis in BMDMs (Figure S3E–H), no difference in sensitivity to chloride channel blockers was observed between WT and KO cells (Figure 3h–j), indicating that VRAC inhibitors likely reduced phagocytosis via LRRC8A‐independent mechanisms.

### 3.4 LRRC8A does not regulate microglial migration and P2RY12‐dependent chemotaxis

**FIGURE 4:**
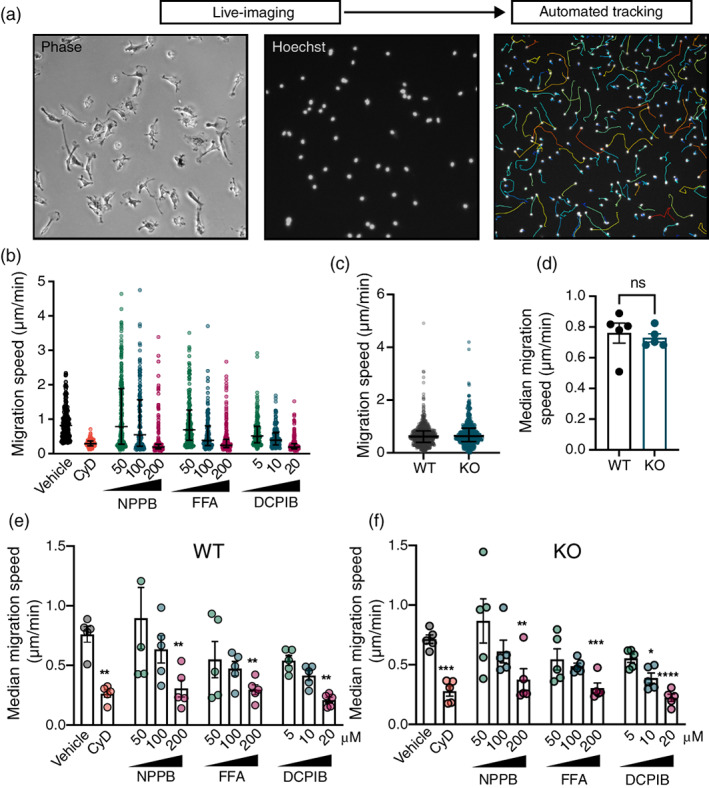
LRRC8A does not regulate microglia motility. (a) Representation of experimental workflow, involving automated tracking of Hoechst‐labeled nuclei in primary adult microglia. (b) Migration speeds of individual wild‐type (WT) microglia from 1 representative mouse treated with either vehicle, Cytochalasin D (CyD; 10 μM), or the chloride channel inhibitors NPPB, FFA and DCPIB at varying doses. (c) Migration speeds of individual microglia from WT and LRRC8A knockout (KO) mice (*n* = 5 mice per genotype) (d) median migration speeds of all microglia from individual WT and KO mice (*n* = 5). (e, f) Median migration speeds of microglia from WT (e) and KO (f) mice treated with vehicle, CyD, or the chloride channel inhibitors NPPB, FFA or DCPIB (*n* = 5). Data presented as median ± IQR (b,c) or mean ± SEM (d–f). Statistical comparisons were made via Student's *t*‐test (d) or one‐way ANOVA with Dunnett's post hoc test (e,f). *p < .05, **p < .01, ***p < .001, ****p < .0001

Volume regulatory processes are proposed to contribute to cell motility and migration by facilitating chloride and water efflux at the trailing edge, and influx at the leading edge of the migrating cell (Schwab et al., 2012). VRAC inhibitors have also been shown to suppress migration and chemotaxis in microglia, neutrophils and a variety of other cell types (Hines et al., 2009; Schwab et al., 2012; Volk et al., 2008). Thus, VRAC has been suggested as a regulator of cell movement. To assess whether LRRC8A deficiency affected cell migratory capacity, we utilized time‐lapse imaging of primary adult microglia which, when cultured with serum, exhibit rapid, spontaneous migration (Bohlen et al., 2017). When analyzing migration, we considered translocation of the cell body, indicative of whole‐cell movement, as opposed to process motility. For this, automated tracking of Hoechst‐labeled nuclei was employed (Figure 4a), which revealed that high doses of VRAC inhibitors (DCPIB, NPPB, FFA) starkly reduced migration speed to a level comparable with the actin depolymerizing agent cytochalasin D (Figure 4b). However, KO cells exhibited no difference in basal migration speed compared to WT cells (Figure 4c,d) and were similarly susceptible to the effects of VRAC inhibitors (Figure 4e,f). Thus, despite an inhibitory quality of chloride channel blockers, VRAC did not appear to contribute to whole‐cell migration in this model.

**FIGURE 5:**
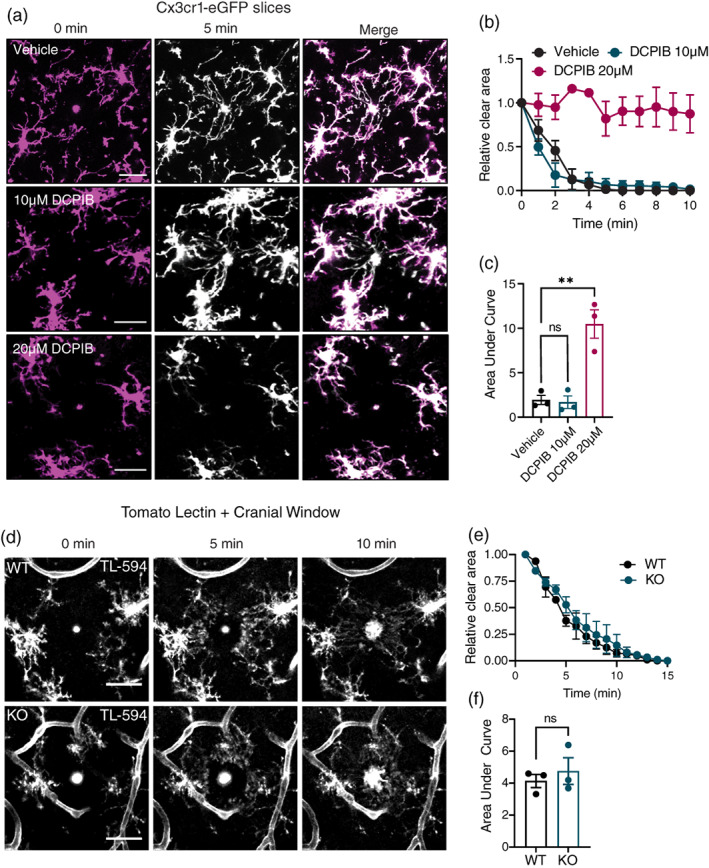
LRRC8A does not contribute to P2RY12‐dependent chemotaxis. (a) Representative Z‐stack projections of Cx3cr1‐eGFP microglia in acute brain slices acquired immediately (0 min) and 5 min after 2‐photon laser ablations were conducted in the presence of either vehicle (DMSO) or DCPIB, scale bars = 25 μm. (b) Chemotaxis of Cx3cr1‐eGFP microglia in the presence of vehicle or DCPIB, quantified as relative process‐free area over time. Average of 4–7 burns per condition from three mice assayed independently. (c) Area under curve quantification of (b). (d) Representative z‐projections of tomato lectin (TL‐594) labeled cortical microglia in wild‐type (WT) and LRRC8A knockout (KO) mice at 0, 5 and 10 min following a 2‐photon laser lesion. Scale bar = 30 μm (e) chemotaxis of WT and KO microglia quantified as total process‐free area over time. Average of 13–14 total burns from three mice per genotype. (f) Area under curve quantification of (e), points represent individual mice. Data are presented as mean ± SEM. Statistical comparisons were performed via one‐way ANOVA with Dunnett's post hoc (c) or Student's *t*‐test (f), **p < .01

The VRAC inhibitors NPPB and tamoxifen have also been reported to suppress microglial P2YR12‐dependent chemotactic responses elicited by focal laser ablations in brain tissue (Hines et al., 2009). To verify this effect on ATP‐dependent chemotaxis, we performed laser ablation injuries in acute brain slices obtained from CX3CR1‐eGFP mice via two‐photon microscopy. We observed consistent chemotactic reactions in control slices, which were inhibited by the presence of high concentrations (20 μM) of DCPIB, but unaffected by moderate concentrations (10 μM; Figure 5a–c). In order to determine whether LRRC8A‐containing VRACs mediated this effect, we performed in vivo ablations in WT and LRRC8A KO mice through implanted cranial windows. Prior to imaging, tomato lectin conjugated to DyLight‐594 was injected into the parenchyma to label microglia. We observed no significant difference in chemotactic response between WT and KO microglia, as quantified by the reduction in process‐free area following laser injury (Figure 5d–f). Thus, both stochastic migration and P2RY12‐dependent microglial chemotaxis appear to occur independently of LRRC8A.

### 3.5 Loss of microglial VRAC does not affect ischaemic damage or microglial morphology after stroke

**FIGURE 6:**
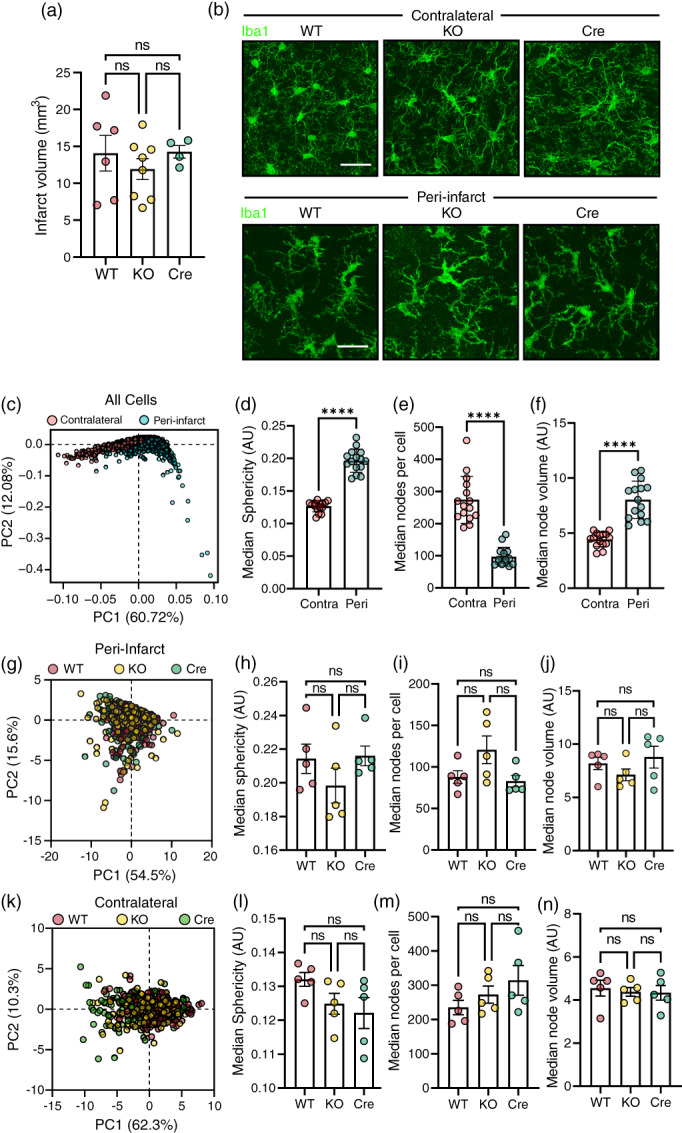
Loss of microglial VRAC does not affect ischaemic damage or microglial morphology after stroke. (A) Infarct volumes from wild‐type (WT), LRRC8A knockout (KO) and Cre mice 24 h after stroke induced by MCA occlusion (MCAO; *n* = 5–8; N.B. one animal experienced partial reperfusion so was excluded from analysis of infarct volume). (b) Representative z‐projections of Iba1‐stained microglia in peri‐infarct and contralateral cortical regions from WT, KO and Cre mice 24 h post‐MCAO. (c) Principal component analysis (PCA) of morphological parameters from all cells (*n* = 1409) quantified in both peri‐infarct and contralateral cortices from all groups of animals (*n* = 15 in total from *n* = 5/group randomly selected from a). (d–f) Median cell sphericity (d), median nodes per cell (e), and median node volume (f) of all measured cells from the contralateral (Contra) and peri‐infarct (Peri) regions. (g) PCA of morphological features of peri‐infarct microglia. (h–j) Median cell sphericity (h), median nodes per cell (i), and median node volume (j) of peri‐infarct microglia from WT, KO and Cre animals (*n* = 5/group). (k) PCA plot of all microglia imaged in the contralateral hemisphere. (l–n) Median cell sphericity (l), median nodes per cell (m), and median node volume (n) of contralateral microglia from WT, KO and Cre animals (*n* = 5/group). Bar graph data are presented as mean ± SEM; data points represent median cell values for individual mice. Statistical comparisons were made via one‐way ANOVA (a, h–j, l–n) or Student's *t*‐test (d–f), ****p < .0001

LRRC8A‐containing VRACs in astrocytes have been shown to contribute to excitotoxic damage in ischemic stroke models by facilitating glutamate release (Yang et al., 2019; Zhou et al., 2020b). We therefore hypothesized that microglial VRACs might also influence ischemic damage, either via transport of neurotransmitters, ATP or other mediators. Alternatively, LRRC8A could have other effects on microglial damage responses which could affect overall tissue injury. To assess this, we performed ferric chloride‐based permanent MCA occlusion on WT, LRRC8A KO and Cre mice. At 24 h post‐occlusion, infarct volumes quantified by cresyl violet staining were comparable between all three genotypes, indicating that microglial VRAC does not influence ischemic damage (Figure 6a). VRAC has also been suggested to support morphological transformation of microglia (Eder et al., 1998). As such, we also assessed microglial morphology in the contralateral and peri‐infarct brain regions (Figure 6b) via confocal microscopy coupled to an automated MATLAB algorithm for extraction of morphological parameters (Heindl et al., 2018). Principal component analysis of the 1409 total microglia analyzed (905 peri‐infarct, 504 contralateral) confirmed that strong differences in microglial morphology were detected between the injured and non‐injured regions (Figure 6c). Specifically, microglia in the peri‐infarct region exhibited significantly higher median sphericity, node volume and significantly fewer skeleton nodes per cell than those in the contralateral hemisphere (Figure 6d–f), corresponding to the expected decrease in process complexity and ramification. However, when analyzed separately, neither the contralateral nor peri‐infarct microglia displayed appreciable genotype‐dependent variability in PCA (Figure 6g,k). Moreover, no significant differences were observed in sphericity, total skeleton nodes per cell, or median node volume between the three genotypes in either the peri‐infarct region (Figure 6h–j) or contralateral cortex (Figure 6l–n). Thus, neither LRRC8A expression nor volume regulatory capacity appear to affect microglial morphology either at baseline or in response to ischemia. Moreover, loss of microglial LRRC8A does not influence ischemic damage.

## 4 DISCUSSION

Volume control and LRRC8A‐dependent chloride conductance have been suggested to regulate microglia and macrophage functions by facilitating cell shape changes which underpin phagocytic, motile and chemotactic behaviors. Since prior studies linking VRAC to microglia/macrophage function date from before the identification of LRRC8‐family members as the molecular components of VRAC, all relied on nonselective pharmacological tools to manipulate channel activities and are difficult to interpret conclusively. We therefore re‐examined these hypotheses using a conditional LRRC8A‐KO model to decipher the true relationship between VRAC, volume regulation and microglial function. Surprisingly, we demonstrate that while RVD is severely compromised in the absence of LRRC8A, the other microglial behaviors we examined remained intact. Indeed, we also observed no apparent impact of LRRC8A‐KO on the survival or morphology of microglia in vivo, and only very subtle effects on gene expression.

These results echo previous findings in astrocytes, which are also capable of normal development, maturation and morphology in the absence of LRRC8A (Yang et al., 2019), implying that VRAC‐dependent volume control such as that observed in vitro under hypotonic conditions is generally redundant for the development and basic functions of most CNS cells. Indeed, not only was mature microglial morphology established without LRRC8A, but these cells were equally capable of transformation to reactive morphologies following cerebral ischemia. As such, we conclude that VRAC conductance is not a major mechanism regulating cell shape changes either at baseline or during CNS injury, though it is possible other chloride channels are involved. It is unclear to what extent microglia require VRAC for volume regulation in vivo, since our data suggest that, at least at baseline and following stroke, loss of LRRC8A does not produce an observable volume regulation deficit in situ despite a clear lack of RVD following hypotonic treatment in vitro. Indeed, to our knowledge no bona fide roles for VRAC in CNS cell volume regulation have so far been demonstrated in vivo. As such, it is possible that VRAC plays redundant roles for volume regulation in a variety of physiological contexts, which are not accurately modeled by current approaches using hypotonic treatment in ex vivo cell cultures. One potential caveat to the model used here is that the Cx3cr1^Cre^ line has been suggested to induce some developmentally‐restricted recombination in non‐microglial cells, including neurons. We observed very similar levels of LRRC8A in the cortex of *Lrrc8a* ^*fl/fl*^ *::Cx3cr1* ^*+/+*^ (WT) and *Lrrc8a* ^*fl/fl*^ *::Cx3cr1* ^*Cre/+*^ (KO) mice, implying that most non‐microglial cells retained LRRC8A expression, but cannot exclude some sporadic loss of LRRC8A in other CNS cells.

Using LRRC8A‐knockout microglia (and macrophages), we show that, contrary to previously published results, VRAC‐dependent ion transport is entirely dispensable for phagocytosis of various substrates. Although we confirm the effect of chloride channel blockers as potent inhibitors of phagocytic activity, our results indicate that this is almost certainly due to activity on other targets, or combined activity on several targets. We did not assess whether microglial phagocytosis is affected by LRRC8A‐KO in situ, though there is no prior evidence to suggest a differential contribution of VRAC to phagocytosis between in vitro and in vivo assays. Nonetheless, given the potential for phenotypic differences between microglia in vivo and in vitro, this remains a potential caveat to the current work.

Since VRAC‐dependent chloride conductance has also been postulated as a mechanism of cell migration, we also assessed stochastic migration of microglia in vitro. LRRC8A‐KO cells exhibited no intrinsic defect in migratory capacity and, importantly, were equally sensitive to the suppressive effect of chloride channel blockers. Taken together, these results contradict the idea that VRAC serves a dual purpose, supporting both osmotic cell shape changes and those required for migration and phagocytosis. Instead, these two processes appear to occur via an entirely different mechanism to RVD. While it is clear that chloride transport plays a significant role in both migration and phagocytosis, the precise channels responsible remain mysterious due to lack of drugs selective enough to target one particular channel, or family of channels.

Due to its importance for microglial‐mediated neuroprotection, we also investigated the involvement of VRAC in P2YR12‐dependent chemotaxis of microglial processes. This mode of directed motility is a critical mechanism by which microglia react to changes in neuronal activity, and can regulate the formation of specialized junctions between microglial processes and neuronal somata, which reduce neuronal calcium load and promote survival under conditions of CNS injury (Cserép et al., 2020). In line with previously published data (Hines et al., 2009), our results confirm that P2YR12‐dependent chemotaxis is abolished by VRAC inhibitors in ex vivo brain slices. However, we demonstrate that VRAC conductance is in fact dispensable for P2YR12‐dependent chemotaxis in vivo. We did not assess whether DCPIB is able to block microglial chemotaxis in the absence of LRRC8A. However, given that LRRC8A‐KO itself did not alter chemotaxis and that DCPIB clearly displays off‐target activity in other assays, it is likely that inhibition of chemotaxis by DCPIB is also VRAC‐independent.

It is possible that chloride channel inhibitors suppress microglial process extension in this model via the same mechanism by which they inhibit stochastic migration and phagocytosis—that is, by producing a widespread block on cell shape changes and movement. However, previous research has suggested that microglia are still capable of extending filopodia during random surveillance whilst exposed to tamoxifen and NPPB at concentrations which inhibit directed motility (Hines et al., 2009). It may therefore be the case that ATP‐induced chemotaxis requires additional chloride transporters distinct from those which support general cell motility.

One potential further question not addressed here is whether or not VRAC contributes to microglial surveillance movements in a similar manner to the two‐pore domain family K^+^ channel THIK‐1 (Madry et al., 2018). Previous studies did not observe any effect of VRAC inhibitors on microglial filopodia extension during undirected surveillance (Hines et al., 2009), but did not assess changes in surveillance behavior on the level of whole cells. Thus, it may be possible that VRAC could contribute to microglial surveillance, though at present there is no direct evidence to suggest this.

Our findings are encouraging in light of recent papers highlighting glutamate‐releasing astrocytic VRAC as a potential therapeutic target for stroke and other CNS injuries due to its role in promoting excitotoxicity (Yang et al., 2019; Zhou et al., 2020b). As with any such strategy, it is important to explore the potential off‐target effects of VRAC inhibition on other CNS cells, particularly microglia, due to their critical role in damage responses. Our results demonstrate that microglia are fully capable of migration, phagocytosis and P2YR12‐dependent chemotaxis in the absence of VRAC, and that abolishing microglial VRAC does not affect the extent of ischemic damage in a mouse model of stroke. Thus, it should be possible to target glutamate transport via astrocytic VRAC in CNS injuries without compromising potential neuroprotective microglial activity. However, another key point raised by our findings is that the current array of VRAC inhibitors are not sufficiently selective for this purpose. Indeed, we and others have demonstrated extensive undesirable off‐target activity in even the current gold‐standard inhibitor, DCPIB (Afzal et al., 2019; Lv et al., 2019; Minieri et al., 2013). As such, the discovery of compounds with greater selectivity for VRAC over other chloride channels would be of great benefit, both to better understand VRAC's roles in physiology and to explore its value as a therapeutic target.

Overall, our results contrast previously suggested roles for VRAC in regulating several important microglial functions. Using a specific LRRC8A conditional knockout model combined with a multifaceted evaluation of microglial function, we have demonstrated that VRAC does not appreciably affect microglial baseline characteristics such as morphology or transcriptional signature, or regulate functional behaviors such as migration, phagocytosis and purinergic chemotaxis. As such, we conclude that VRAC does not critically regulate microglial functions of relevance to brain injury such as ischemia.

## CONFLICT OF INTEREST

The authors declare no conflict of interest.

## Supporting information


**Figure S1** **– Cx3cr1** ^**Cre**^ **does not affect LRRC8A expression or RVD:**
**(A‐B)** Western blots showing expression of LRRC8A protein in WT and Cx3cr1^Cre/+^ (Cre) microglia (**A**) and BMDM (**B**) (representative of n = 3 mice). **(C‐D)** Fluorescence traces **(C)** of calcein‐loaded microglia from WT and Cre mice exposed to hypotonic (218 mOsm/kg) conditions at 5 minutes, quantified as area‐under‐curve in **(D)** (n = 3 mice), presented as mean ± SEM.

**Figure S2** **– Expression of chloride channel genes in microglia:**
**(A)** Expression of each member of the LRRC8 (VRAC), Anoctamin (TMEM16), Chloride intracellular channel (CLIC), Voltage‐gated chloride channels (CLCN), Tweety (TTYH) and bestrophin (BEST) families of chloride channels in acutely isolated microglia from WT, LRRC8A‐KO (KO) and Cx3cr1^Cre/+^ (Cre) mice, assessed by RNA‐Seq (n = 3). No significant differences were present between genotypes for any gene, as assessed by differential expression analysis in DESeq2. **(B‐C)** Expression of exons 1–2 **(B)** and exon 3 **(C)** of the *Lrrc8a* gene in WT and KO microglia, as assessed via qPCR (n = 4 mice). Expression values are all normalized to the housekeeping gene *Gapdh*, and those of exon 3 are further normalized to abundance of exon 1–2 in the same sample. Data presented as mean ± SEM, statistics in **B‐C** were conducted via Student's *t*‐test, ** *p* < 0.01.

**Figure S3** **LRRC8A is dispensable for phagocytosis in macrophages (A)** Phase‐contrast and fluorescent photomicrographs showing uptake of pHrodo‐tagged IgG‐opsonised red blood cells (RBC) in wild‐type (WT) and LRRC8A‐knockout (KO) bone marrow‐derived macrophages (BMDM). Scale bar = 30 μm. **(B‐D)** Phagocytic uptake curves and area under curve quantification of WT and KO BMDM exposed to pHrodo‐IgG‐RBC **(B)**, pHrodo‐Zymosan **(C)**, or pHrodo‐*E. Coli*‐derived bioparticles **(D). (E‐G)** Phagocytic index curves of primary BMDM exposed to pHrodo‐*E. Coli* bioparticles in the presence of vehicle (Veh; DMSO), Cytochalasin D (CyD) or NPPB **(E)**, FFA **(F)** or DCPIB **(G)**. **(H)** Area under curve quantification of E‐G. Data are presented as mean ± SEM. Individual data points in AUC graphs represent biological replicates. Phagocytic index graphs are mean ± SEM of one experiment containing 2–3 biological replicates. Statistical comparisons in B‐D were made via Student's *t*‐test and in H via one‐way ANOVA. * p < 0.05, ** p < 0.01, *** p < 0.001, **** p < 0.0001.

## Data Availability

The data that support the findings of this study are available from the corresponding author upon reasonable request.
